# Evaluation of whole genome sequencing protocols and target-capture panels for detection of respiratory viruses in clinical specimens

**DOI:** 10.3389/fmicb.2026.1964559

**Published:** 2026-10-23

**Authors:** Daniel Kelly, Ketharani Yogananthan, Priya Subramaniam, Tiina Talts, Carmen F. Manso, Beatrix Kele, David F. Bibby, Mark Zuckerman, Jean L. Mbisa

**Affiliations:** 1 Virus Reference Department, UKHSA, London, United Kingdom; 2 South London Specialist Virology Centre, King’s College Hospital NHS Foundation Trust, London, United Kingdom

**Keywords:** clinical diagnostics, quantitative PCR, rapid multiplex PCR, respiratory virus, whole genome sequencing (WGS)

## Abstract

Respiratory viruses are major contributors to global morbidity and mortality. When viral mutations compromise detection by conventional diagnostic assays, identifying cases and tracing transmission becomes challenging. Whole genome sequencing (WGS) improves on conventional diagnostics as it can detect viruses with novel mutations. This study evaluated the performance of the ePlex, a rapid multiplex polymerase chain reaction (PCR) assay (RMPA), an in-house real-time reverse transcription PCR assay (RT-qPCR) and WGS for detecting respiratory virus infections in clinical specimens. Sixty-one sequence libraries were prepared, and bait enriched from nasopharyngeal swabs, collected in 2021, which were negative for SARS-CoV-2 but positive for co-infections of respiratory viruses detected by the ePlex. An in-house bioinformatics pipeline enabled a metagenomic analysis approach to be compared with the ePlex and RT-qPCR. In most swabs, respiratory viruses were detected by WGS (average genome recovery: 85.9%) or RT-qPCR which concurred with the ePlex result, but as single viruses rather than co-infections. WGS recovered respiratory syncytial viruses (80%) and metapneumoviruses (70%) most often. This study demonstrates the power of WGS and bait capture in resolving the genomes of respiratory pathogens from limited clinical material. Detection of a single virus, rather than co-infections, in this instance could highlight the targeted nature of bait capture technology or competition between viruses during bait hybridization. The number of respiratory syncytial viruses detected (16/20) could be due to a spike in activity detected after the alleviation of non-pharmaceutical interventions in the COVID-19 pandemic.

## 1 Introduction

Respiratory viruses have been responsible for at least half of major viral outbreaks during the twenty-first century (Casadevall, 2024). The emergence of novel pathogens, together with the ongoing burden of endemic viruses, highlights the importance of rapid and reliable molecular diagnostics. Early detection of infected individuals supports transmission-control measures and informs public health responses. The effectiveness of a diagnostic tool depends largely on its turnaround time, accuracy and sensitivity, all of which contribute indirectly to the success of transmission-control strategies.

Real-time PCR (qPCR) is a commonly used method for molecular diagnosis of infectious disease. This technique amplifies a specific genomic target and generates a fluorescent signal proportional to the amount of amplified product. Consequently, samples with lower concentrations of viral nucleic acid require a greater number of amplification cycles to reach the detection threshold. Despite its high analytical performance, assay sensitivity and specificity can be influenced by sample, quality, assay design, workflow execution and data interpretation (Bustin, 2024).

Rapid multiplex PCR assays (RMPA) use similar amplification chemistry while providing commercially available, highly standardised and user-friendly diagnostic platforms. Examples include Biofire (bioMérieux), ePlex (Roche), Xpert Xpress (Cepheid) and QIAstat (Qiagen) for which panels are available to detect multiple pathogens. Unlike QIAstat, Biofire, ePlex and Xpert Xpress generally provide a qualitative rather than quantitative output. However, investigation of false-negative or false-positive results can be challenging because assay chemistry is often proprietary and amplification curves are not always accessible to the user.

WGS also enables metagenomic analyses and can be enriched for pathogens of interest through RNA/DNA bait technology (Alleweldt et al., 2021). In the United Kingdom, scale up of whole genome sequencing (WGS) was observed at the beginning of the SARS-CoV-2 pandemic with the formation of the Coronavirus Disease 2019 Genomics UK Consortium (COG-UK). COG-UK applied WGS to infected individuals across the UK which would map out transmission patterns and the emergence of variants at high resolution and inform public health policy (The COVID-19 Genomics UK (COG-UK) consortium, 2020).

Several studies exist in the literature where clinical evaluations have been performed between RMPAs and qPCR (Steiner et al., 2021; Boers et al., 2020; Mills et al., 2024). Two studies noted a loss in sensitivity when using clinical samples with viral cycle threshold (Ct) values above 30 (Steiner et al., 2021; Nijhuis et al., 2017). However, most evaluations are made between cartridge-based assays of different manufacturers, and to a lesser extent with qPCR-based assays (Steiner et al., 2021; Mills et al., 2024). Two studies comparing the performance of the BioFire Pneumonia Panel RMPA and WGS exist in the literature, one uses bronchoalveolar lavage fluid specimens and the other nasophayngeal swabs (Kling et al., 2024; Kapel et al., 2023). However, to our knowledge, a comparison between WGS, an inhouse RT-qPCR and an RMPA for nasopharyngeal swabs has not been described in the literature.

In this study, the efficiency of three different library preparation and bait capture protocols was investigated against six respiratory pathogens. The most sensitive protocol was applied to nasopharyngeal swabs, which via testing by the ePlex were selected due to the presence of viral co-infections excluding SARS-CoV-2. Metagenomic bioinformatic analyses and multiplex RT-qPCR assays were used to evaluate agreement between these workflows and the ePlex.

## 2 Materials and methods

### 2.1 Preparation of virus isolates and clinical sample collection

To optimise the WGS workflow, clinical samples containing adenovirus genotype 14, influenza A (H1N1), influenza A (H3N2), respiratory syncytial virus (RSV) A, RSV B and SARS-CoV-2 were selected for virus isolation. Viruses were propagated in cell culture, except for SARS-CoV-2, which was extracted directly from a clinical sample. RSV and adenovirus genotype 14 were passaged in Hep-2 cells maintained in Eagle’s Minimum Essential Medium (EMEM) supplemented with 10% foetal bovine serum (FBS), while influenza viruses were propagated in embryonated hen eggs using standard protocols (World Health Organization, 2011). Viral nucleic acids were extracted from the cultural isolate or clinical specimen using the Chemagic 360 Platform (Perkin Elmer, 2024-0020) and serially diluted in nuclease free water (Table 1).

**Table 1:** Ct values of respiratory targets in tissue culture system extracts for library preparation and RNA bait capture.

Respiratory virus	Isolate	Dilution		
		Neat	1:10	1:100
Adenovirus	Adenovirus type14 sample p69 22/10/2011	19.44	23.27	26.45
Influenza (H1N1)	A/California/7/2009	18.14	21.73	25.34
		18.65	22.11	25.31
Influenza (H3N2)	A/Kansas/14/2017	16.39	20.05	23.45
		17.81	21.25	24.59
RSV A	RSV-A LONG Batch: 110515	16.39	19.77	22.84
RSV B	RSV-B 9320 Batch: 110515	17.91	20.55	23.43
SARS-CoV-2	RE2100158	23.94	27.18	30.38
		23.85	27.12	30.35

For the WGS, ePlex and semi-quantitative RT-qPCR workflows, the sample size was determined by sample availability. A total of 61 nasopharyngeal swabs collected in 2021 and stored in viral transport medium (VTM) met the inclusion criteria. Between testing procedures, primary samples were stored at −80 °C and underwent freeze thaw-cycles one and two prior to WGS and RT-qPCR testing, respectively.

### 2.2 Optimisation of WGS workflow

For the SMARTer Stranded Total RNA-Seq Kit v2 [SST] (Takara Bio, 634,414) combined with the Respiratory virus oligo panel v2 [RVOP] (Illumina, 20,044,312) or the Respiratory virus research panel [RVRP] (Twist Bioscience, 102,937) library preparation was performed as described by Manso and colleagues with the exception that no DNase digestion step was performed (Table 2) (Manso et al., 2020). For the Twist library preparation EF kit 2.0 [TLP] (Twist Bioscience, 104,207) combined with the RVRP library preparation was performed as described by the manufacturer (Table 2). For all combinations, bait capture was performed as described by the manufacturer.

**Table 2:** Combinations of library preparation and sequence capture.

Label	Library preparation	RNA bait capture
SST-RVOP	SMARTer Stranded Total RNA-Seq Kit v2 (Takara)	Respiratory virus oligo panel
SST-RVRP		Respiratory virus research panel
TLP-RVRP	Twist library preparation kit (TLP)	

Prior to bait capture, indexed libraries were normalised by volume and grouped to ensure the pool mass was within the limits of enrichment capacity. For the SST-RVOP, concentration of the indexed libraries was achieved by adding two times the volume of AMPure beads (Beckman Coulter, A63881), performing two 80% ethanol washes and eluting into 2.5 μL of nuclease free water. Otherwise, concentration was performed as described by the manufacturer. Bait enrichment output for each combination was normalised by mass and each respective 2 nM pool was run on the NextSeq 1,000 Sequencing System (Illumina, 20,038,898) with NextSeq 1000/2000 Reagents (Illumina, 20,050,264) in separate sequencing runs.

### 2.3 WGS workflow

In total, 150 μL of swab VTM underwent nucleic acid extraction on the NucliSENS easyMAG (BioMérieux, 200,110) extraction platform using NucliSENS easyMAG (BioMérieux, 280,130–280,134) extraction reagents with the “Generic 2.0.1” protocol. In parallel, three aliquots of negative human plasma and five aliquots of nuclease free water were designated as no template controls (NTC) and underwent extraction. The total volume of nucleic acid eluate for each extract, 25 μL, was treated with the SST-RVOP combination as described previously but samples were normalised by mass rather than volume. Bait enrichment output was normalised by mass, and a 2 nM pool was run on the NextSeq 550 System (Illumina, SY-415-1002) with the NextSeq500/550 High Output Reagent Cartridge v2 kit (Illumina, 20,024,908).

### 2.4 ePlex workflow

In total, 200 μL of swab VTM was added to a Cobas ePlex respiratory pathogen panel 2 cartridge (Roche, 09555641001) and tested on the Cobas ePlex system (Roche, 10,107,545,001 and 10,027,762,001). The test was performed as described by the manufacturer.

### 2.5 Semi-quantitative RT-qPCR workflow

In total, 150 μL of swab VTM and 150 μL of lysis buffer underwent automated extraction on the Chemagic 360 (Revvity, 2024-0020) and 50 μL of total nucleic acid extract was eluted. A Semi-quantitative (Taqman) inhouse RT-qPCR was performed on a QuantStudio 7 Flex Real-Time PCR System (Thermo Fisher Scientific, 4,485,688). Each multiplex final reaction contained primers and probes to target the viruses (Supplementary Table S2), 1x TaqPath 1-Step Multiplex Master Mix (Thermo Fisher Scientific, A28525) and 5 μL of nucleic acid extract to a total volume of 20 μL. Semi-quantitative RT-qPCR cycling conditions were as described in the laboratory developed protocol (Supplementary Table S2).

### 2.6 Data analysis

For the RT-qPCR workflow, a virus detected in a swab was included for subsequent analyses if it had a Ct value below 35. For the WGS workflow, virus detected in a swab was included in subsequent analyses if reads were absent from the NTCs and aligned to reference sequences from the corresponding virus groupings: Adenovirus, Coronavirus, Enterovirus, Influenza, RSV, Metapneumovirus and Parainfluenza virus. Rhinoviruses were included within the Enterovirus group.

NextSeq output was interleaved and paired reads were extracted with the khmer (Crusoe et al., 2015), and dehumanised by alignment to GRCh38.p14 with Burrows-Wheeler Aligner (BWA) (Li and Durbin, 2009). Reads were then depth-normalised using khmer, to remove similar reads and reduce redundancy whilst maintaining read diversity. Reads were then filtered by quality via Trimmomatic and *de novo* assembled with metaSPAdes (Nurk et al., 2017; Bolger et al., 2014). If an assembled sequence was >150 bases, it was queried against the RefSeq viral database (accessed: 24/6/24) with BLASTN (Altschul et al., 1990). Where a match was identified, quality-filtered reads (without depth normalisation) were aligned to the reference with the highest Expect value. Read mapping statistics were derived from SAMtools and QuasiBAM (Li et al., 2009; Penedos et al., 2015).

On target reads (OTRs) were defined as the percentage of reads which aligned to the reference sequence derived from the RefSeq viral database. Virus genome recovery was defined as the breadth of coverage provided by swab reads toward the reference derived from the RefSeq viral database. A near-complete genome was defined as virus genome recovery > = 95%. A suspected co-infection was defined as detecting multiple different viruses in the same swab, where: (a) at least 75% of each genome was recovered or (b) each Ct value was <30.

For the optimisation experiments, statistical comparisons were performed using paired Wilcoxon-signed-rank tests in R with Bonferroni correction for multiple testing. For each workflow, positive percent agreement (PPA) and negative percent agreement (NPA) were calculated, and agreement was assessed using Cohen’s kappa.

## 3 Results

### 3.1 Evaluation of library preparation kit and bait enrichment strategy

For undiluted extracts, significant median fold changes of 7.4, 9.4 and 1.4 were observed between SST/RVOP vs. SST/RVRP, SST/RVOP vs. TLP/RVRP and SST/RVRP vs. TLP/RVRP (Figure 1). For the 1:10 extracts, an increase of 1.9 between SST/RVOP and SST/RVRP and a decrease of 2.5 between SST/RVRP and TLP/RVRP (Figure 1). For the 1:100 extracts, changes mirrored the undiluted extracts and decreases of 8.5, 71.5 and 6.8 were observed between SST/RVOP vs. SST/RVRP, SST/RVOP vs. TLP/RVRP and SST/RVRP vs. TLP/RVRP (Figure 1). For the diluted extracts, the median number of mapped reads was always lower if TLP was used rather than SST (Figure 1). If the SST/RVRP or TLP/RVRP was used, the range of median values across dilution points was more pronounced (1.6 × 10^3^ to 5.9 × 10^1^ RPM or 2.8 × 10^3^ to 5.9 × 10^0^ RPM, respectively).

**Figure 1:**
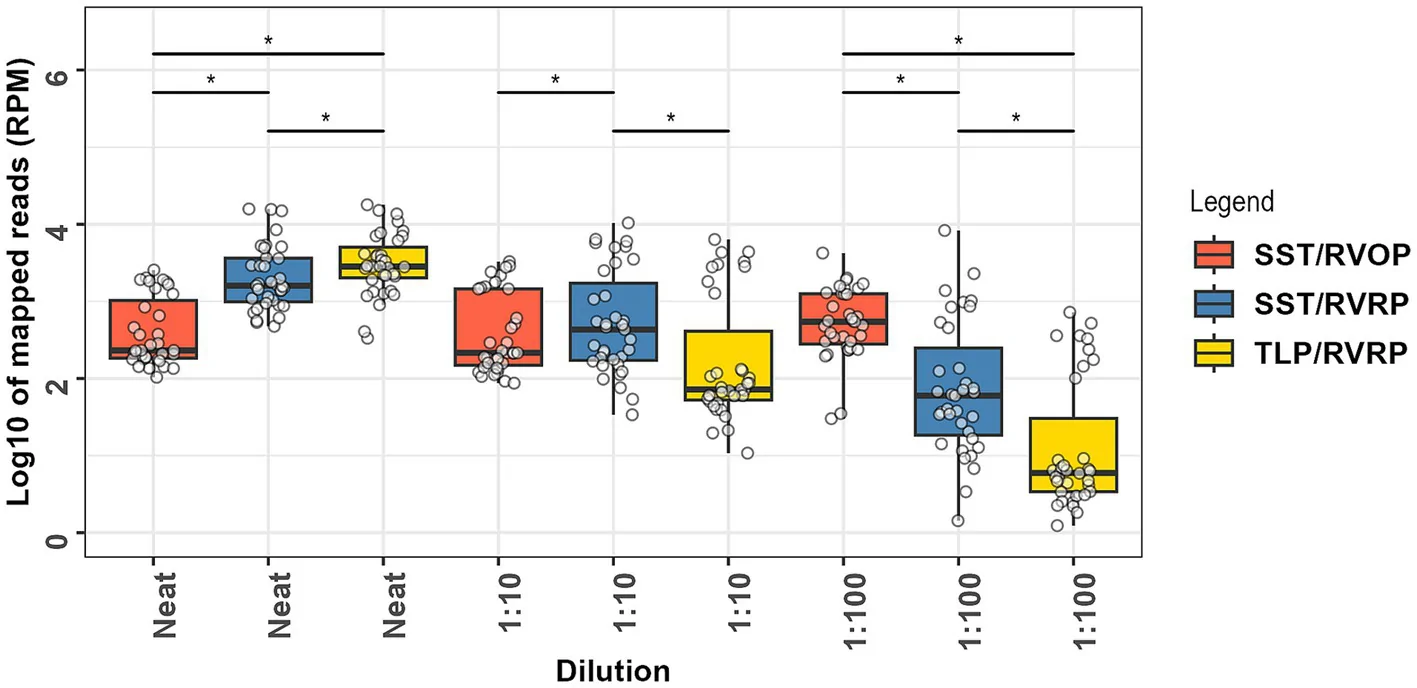
Number of reads mapping to adenovirus, H1N1, H3N2, RSV A, RSV B and SARS-CoV-2 through different combinations of library preparation and bait capture.

### 3.2 Identifying swab constituent viruses

For the RT-qPCR workflow, bocavirus and parainfluenza virus were not measured. For the RT-qPCR and WGS workflows, virus detection plexity maxed out at three but the ePlex workflow was able to detect four different viruses. Among the swabs designated as coinfections by the ePlex, dual-virus co-infections accounted for 63.9% of the collection. For the RT-qPCR and WGS workflows, there were instances where no virus was detected in 14.8 and 26.2% of swabs, respectively (Figures 2A,B). Each workflow RT-qPCR and WGS most frequently identified a single virus, accounting for 57.4 and 60.7% of samples, respectively. This pattern remained true if swabs containing bocavirus or parainfluenza virus were excluded from all analyses (data not shown). In contrast to the ePlex assay, observations of multiple different viruses in the same swab were in the minority for the RT-qPCR and WGS workflows at 27.9 and 13.1% of the swabs, respectively (Figures 2A,B).

**Figure 2:**
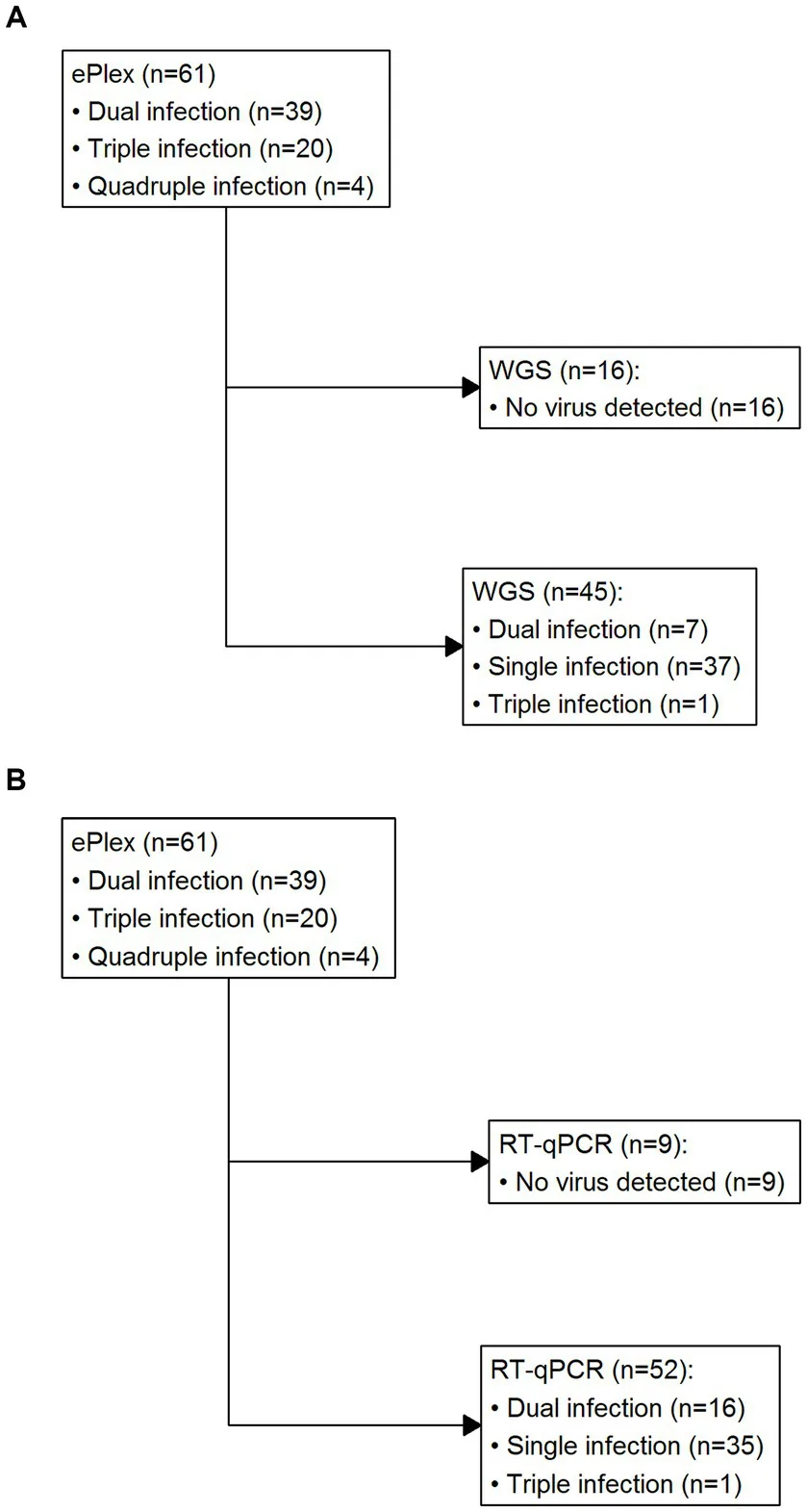
**(A)** Number of viruses detected in each swab if the ePlex and RT-qPCR workflows were compared. **(B)** Number of viruses detected in each swab if the ePlex and WGS workflows were compared.

Among samples containing multiple viral detections, enterovirus was the pathogen most frequently associated with co-detections identified by ePlex and RT-qPCR, whereas parainfluenza virus predominated among co-detections identified by WGS.

### 3.3 Comparisons between the workflows

The ePlex assay generated qualitative results, whereas WGS and RT-qPCR provided quantitative measures that enabled assessment of concordance between the diagnostic workflows. The ePlex and RT-qPCR workflows detected 49 viruses which were not found by the WGS workflow. The Ct values of these viruses ranged from 23.4 to 39.9 and nearly two thirds of these were enterovirus (Figure 3).

**Figure 3:**
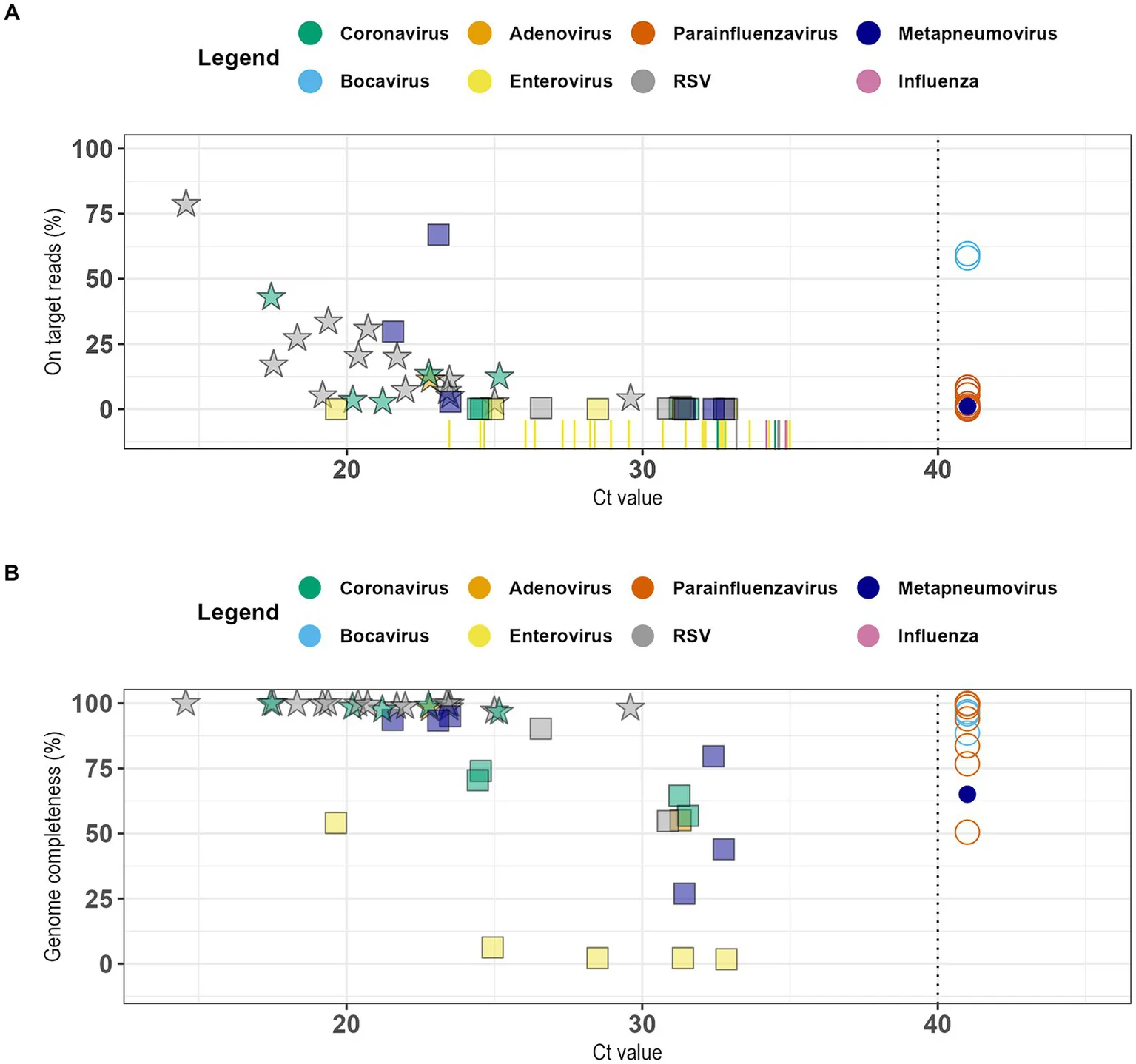
**(A)** Number of on-target reads versus Ct value for each virus type (star= > 95% genome recovery, square = <95% genome recovery, circle = not detectable by RT-qPCR or no RT-qPCR assay available and line = detected by RT-qPCR but no reads found by whole genome sequencing). **(B)** Genome completeness versus Ct value for each virus type (star= > 95% genome recovery, square = <95% genome recovery, circle = not detectable by RT-qPCR or no RT-qPCR assay available and line = detected by RT-qPCR but no reads found by whole genome sequencing).

For all three workflows, there were 40 instances where virus detections agreed. Most of these instances had on target reads (OTRs) below 50% and a Ct value below 30 (27/40). In half of the instances, a near complete genome was recovered and most of these were either coronavirus or RSV. Beyond the Ct value of 30, if enterovirus was excluded, at least 50% of the genome was recovered in half of the instances even though OTRs were below 1% (Figure 3). In all four instances, where enterovirus was detected by the WGS workflow, the enrichment appeared to underperform with a decrease in genome completeness of at least 25% when compared to other viruses at similar Ct values.

In total there were multiple parainfluenza virus and bocavirus detections and a single metapneumovirus detection which were not tested or not found by the RT-qPCR workflow. Detections of bocavirus had high numbers of OTRs in contrast to parainfluenza virus and the single metapneumovirus (Figure 3). But in this grouping of viruses that were not tested or not detected by RT-qPCR, at least half of the virus genome was recovered in most instances (Figure 3).

The ePlex workflow can type seasonal coronavirus, influenza, parainfluenza and RSV, and for all the swabs tested, every virus detected by the RT-qPCR or WGS workflow was found by the ePlex and there was complete agreement in typing (data not shown).

If the WGS or RT-qPCR workflows were compared to the ePlex, positive percent agreement (PPA) was 100% for all viruses tested (Figure 4). And for WGS or RT-qPCR versus the ePlex, overall percent agreement (OPA) was highest for metapneumovirus, at 70.00% (39.68–89.22) or 60.00% (31.27–83.18), and RSV, at 80.00% (58.40–91.93) or 85.00% (63.96–94.76), respectively (Figure 4). OPA was lowest for adenovirus and enterovirus at 12.50% (3.50–36.02%) or 31.25% (14.16–55.60%) and 11.76% (4.67–26.62) or 32.35% (19.13–49.16), respectively (Figure 4). If the WGS and RT-qPCR workflows were compared, the OPA was highest for metapneumovirus, at 90.00% (59.59–98.21), and RSV, at 95.00% (76.39–99.11), and for 7/8 viruses tested the OPA was higher than the PPA (Figure 4).

**Figure 4:**
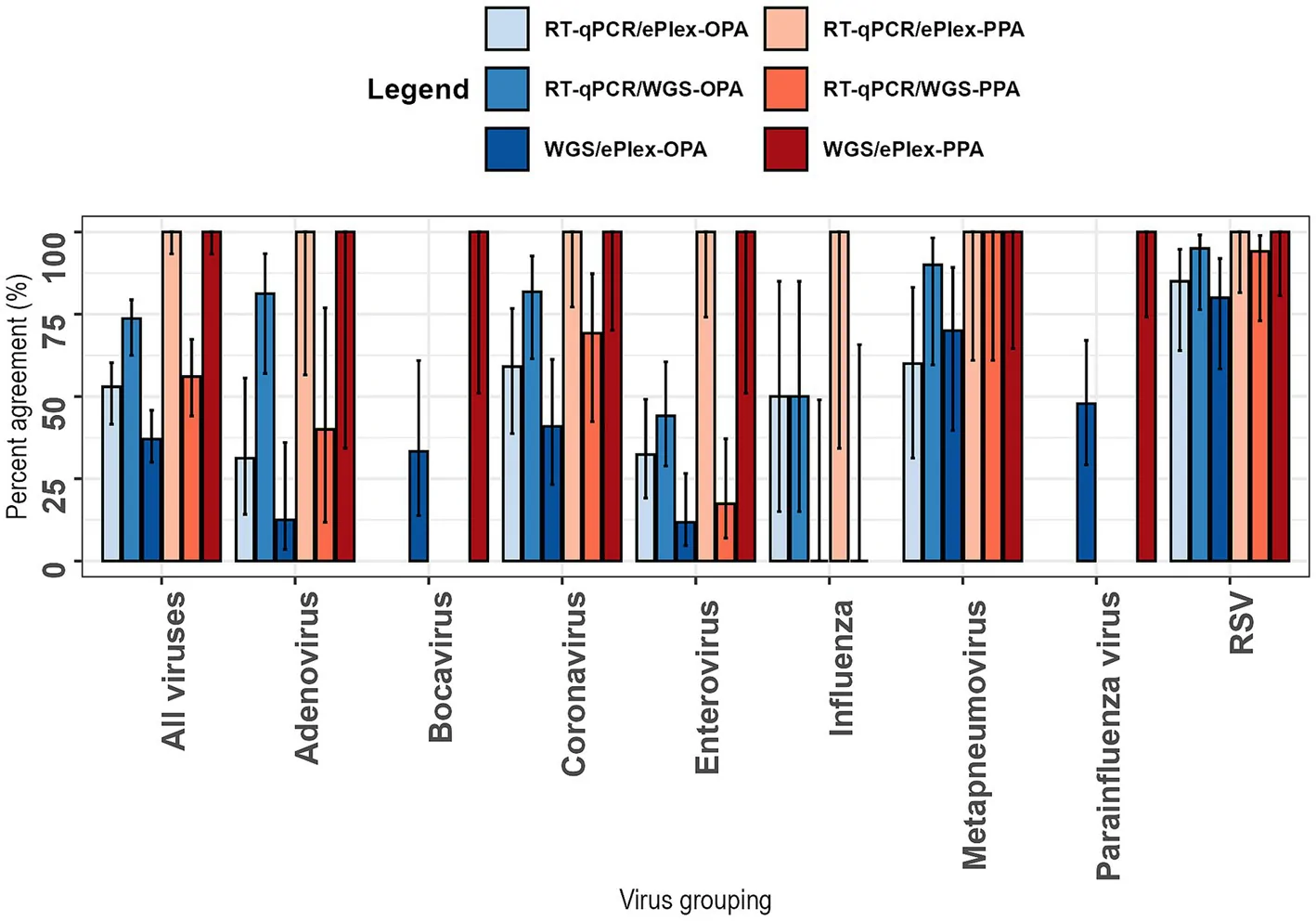
Positive percent agreement and overall percent agreement for each virus grouping (error bars are the 95% confidence intervals).

For all viruses, OPA was greater for RT-qPCR versus WGS, at 73.70% (62.48–79.40), when compared to RT-qPCR versus ePlex, at 52.95% (41.56–60.26), or WGS versus ePlex, at 37.04% (30.02–45.81) (Figure 4). For the RT-qPCR and WGS workflows there was moderate agreement in virus detection (κ = 0.47, *p* = <0.05). Moderate agreement existed for adenovirus (κ = 0.48, *p* = <0.05) and substantial agreement for coronavirus (κ = 0.65, *p* = <0.05), RSV (κ = 0.83, *p* = <0.05) and metapneumovirus (κ = 0.78, *p* = <0.05).

### 3.4 WGS workflow genome recovery and identifying swab co-infections

By comparing genome recovery of each virus type to the recovery of all virus sequences, it was possible to identify where capture over or underperformed. Relative to all the viruses captured, the genome recovery of bocavirus, parainfluenza virus and RSV was greater than the average. Genome recovery of coronavirus and metapneumovirus was near to the average whilst adenovirus and enterovirus genome capture underperformed (Figure 5). The underperformance of enterovirus capture is highlighted by the significant difference (*p* < 0.05) between enterovirus genome recovery when compared to all the viruses detected by the WGS workflow (Figure 5).

**Figure 5:**
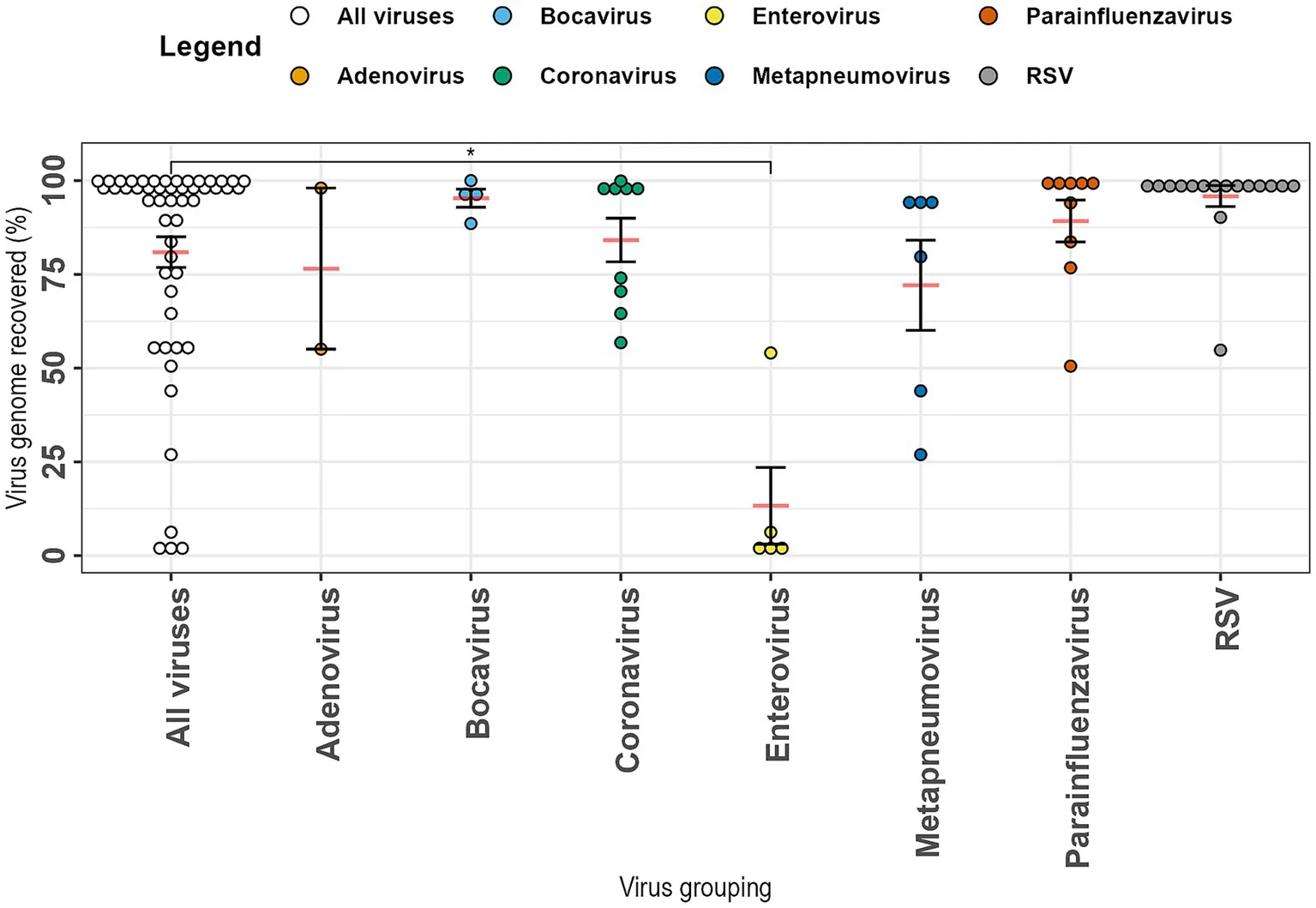
Percentage of virus genome recovery grouped by virus type (red bar = mean, error bars = standard error).

Twenty-one swabs where multiple viruses were identified by the RT-qPCR or WGS workflows which agreed with the ePlex were analysed further. In 71.4% of the swabs in this grouping, over 70% of a single virus genome was recovered. Applying the study-specific operational criteria, two of the swabs fulfilled both criteria, these two swabs constituted of RSV-A and metapneumovirus or enterovirus, parainfluenza virus and RSV-B.

## 4 Discussion

Respiratory-virus infection is diagnosed most often using nasopharyngeal swabs analysed by antigen-based, e.g., point of care lateral flow test or molecular diagnostic assays. In this study, an optimised bait-enrichment workflow was developed by evaluating multiple library-preparation and target-enrichment strategies and combined with an unbiased metagenomic analysis.

The SST/RVRP or TLP/RVRP outperformed the SST/RVOP in samples where the concentration of viral nucleic acid was high, however, this trend was reversed if the amount of viral nucleic acid decreased, with SST RVOP read recovery being least variable across all dilution points. Four major differences exist between the protocols evaluated. Firstly, the SST is designed for small and fragmented RNA inputs and hence a fragmentation step is absent, secondly, adapters are added via PCR which can increase the representation of less abundant nucleic acids unlike the TLP where ligation is used. Thirdly, the SST utilises a ribosomal RNA depletion step, which would increase the representation of viral targets. Lastly, there are differences in the bait capture pools of the RVRP, which is designed via compact aggregate of targets for comprehensive hybridization (CATCH), and RVOP which has led to less baits in the latter (41,047 versus ~7,800) and is likely due to mismatch tolerance (Metsky et al., 2019). A lesser number of baits in the pool could lead to an increased probability of bait-viral target interaction and this could explain the higher ready recovery at lower nucleic acid concentrations.

Optimisation of this methodology had two limitations: due to limited resources it was necessary to attempt to capture both DNA and RNA viruses in a single experiment. This meant DNase digestion was not performed prior to library preparation and host, or bacterial contaminants were not depleted. Also, for the initial evaluation of capture methods, the viruses tested were grown in a tissue culture system and then nucleic acids extracted and diluted. Differences in the composition between a nasopharyngeal swab and a tissue culture system could explain the differences in adenovirus sequence recovery. DNA viruses (adenovirus and bocavirus) were recovered from the swabs at a lower rate when compared to the RNA viruses, and this could be due to the DNA-dependent DNA polymerase activity of the reverse transcriptase from the SST or the detection of expressed viral RNA.

The ePlex was able to detect the most viruses per swab and at a higher rate when compared to the RT-qPCR and WGS workflows. If viruses per swab were compared between the RT-qPCR and WGS workflows, one virus per swab was most common for both. For WGS, although one virus per swab was most common, this could be due to the dominant pathogen outcompeting the other for reagents during library preparation and capture, leading to masking of the presence of other viruses. The detection of multiple viruses by an RMPA followed by identification of only a single virus through bait enrichment and sequencing is not uncommon (Kapel et al., 2023). This observation in combination with ePlex detections in clinical specimens, which were not detected by RT-qPCR and WGS, places uncertainty upon these detections, and whether it is clinical infection or carriage. In this study, conservative criteria were applied to define co-infections, and 2/61 swabs fulfilled them, with one constituting enterovirus, RSV and parainfluenza virus and the other RSV and metapneumovirus. Criteria were based upon publications measuring the success of infection in a tissue culture system at different Ct values from RT-qPCR. For Influenza A and B virus, a 50% chance of infection was observed at Ct values 31.1 and 31.8 and for SARS-CoV-2, 19.4% at Ct value 31 (Jaafar et al., 2021; Saygan et al., 2026).

Through the WGS workflow the highest number of near-complete genomes were detected for RSV (14/16) which could be due to the lack of diversity in RSV genomes but also could be due to the timing of swab collection which coincided with a spike in RSV activity in the northern hemisphere (Ujiie et al., 2021; Quintero-Salgado et al., 2024; Kume et al., 2025). Enterovirus demonstrated notably poorer capture performance than other virus groups evaluated. Detection by WGS was substantially lower than RT-qPCR (12% versus 74%), and near-complete genomes were rarely recovered, even at Ct values below 30. This observation is consistent with previous studies that reported lower sequence-capture efficiency for enteroviruses (Child et al., 2023). Given the considerable genetic diversity of enteroviruses, the reduced recovery may reflect incomplete representation of this diversity within the RVOP probe set (Royston and Tapparel, 2016). However, there was one exception where an incomplete metapneumovirus genome was recovered by the WGS workflow, but it was not detected by RT-qPCR. One of the coverage gaps, between positions 827 and 922, overlapped the binding sites of the primer and probe and therefore would have contributed to the loss in signal observed. The loss of RT-qPCR signal demonstrates an advantage of the sequence capture approach, where it’s use in surveillance is not constrained by virus diversity but can inform on the design of sequence specific diagnostic assays. Further confirmatory experiments should design primers to target conserved regions flanking this region and determine the sequence by Sanger sequencing.

Overall, RT-qPCR versus WGS had a higher OPA in comparison to RT-qPCR versus the ePlex or WGS versus the ePlex, and this observation was confirmed by moderate agreement via Cohen’s kappa. OPA for all viruses was lowest for the WGS versus ePlex for which poor genome recovery of adenovirus and enterovirus contributed to. Several viruses had a lower PPA than OPA if the RT-qPCR workflow was compared to WGS which could indicate lower sensitivity of WGS relative to RT-qPCR and coincides with a loss in signal being associated with high Ct values (12/35 Ct value <30).

Evaluation of the individual workflows was limited by sample availability, resulting in a relatively small and potentially biassed clinical sample set, and meant NPA could not be calculated for the ePlex workflow against WGS or RT-qPCR. Furthermore, comparisons between workflows may have been affected by swabs undergoing freeze–thaw cycles, for which the effects can vary between different viruses. Freeze–thaw cycling can lead to the loss of an intact capsid, degradation of the virus genome and therefore influence virus nucleic acid recovery.

The increasing accessibility of next-generation sequencing platforms has expanded opportunities for metagenomic investigation of respiratory infections. WGS provides a flexible and scalable approach that is less constrained by viral genetic diversity and can support both surveillance and diagnostic development. As sequencing technologies become increasingly automated and accessible, capture-based metagenomic sequencing is likely to play an expanding role in respiratory virus diagnostic and public health surveillance.

## Data Availability

The datasets presented in this study can be found in online repositories. The names of the repository/repositories and accession number(s) can be found at: https://www.ncbi.nlm.nih.gov/, accession numbers PZ795375 - PZ795377, PZ797549 - PZ797567.
