# Supplementary material for: Evaluation of whole genome sequencing protocols and target-capture panels for detection of respiratory viruses in clinical specimens
Source: Front Microbiol. 2026 Oct 23;17:1964559. doi: 10.3389/fmicb.2026.1964559 (PMC13739801; doi:10.3389/fmicb.2026.1964559)
Supplement: Supplementary file 1 [file Table_1.docx]

Supplementary Material

# 1 Supplementary Figures and Tables

## 1.1 Supplementary Tables

**Supplementary Table 1:** RT-qPCR and WGS metrics for each virus detected (ND=not detected, NT=not tested).

| Virus | Ct | OTR | Reads | Complete | Pt | Reference sequence | Accession number |
| --- | --- | --- | --- | --- | --- | --- | --- |
| Coronavirus | 31.55 | 0.05 | 1962162 | 56.80 | Pt1 | NC_006213.1 |  |
| RSV | 20.39 | 20.31 | 1962162 | 99.80 | Pt1 | NC_001781.1 | PZ797549 |
| Adenovirus | ND | 0.00 | 1962162 |  | Pt1 |  |  |
| RSV | 33.18 | 0.00 | 2069242 | 0.00 | Pt2 |  |  |
| Coronavirus | 21.21 | 2.73 | 2069242 | 97.47 | Pt2 | NC_006213.1 | PZ797553 |
| Enterovirus | ND | 0.00 | 2069242 | 0.00 | Pt2 |  |  |
| Parainfluenzavirus | NT | 8.38 | 901272 | 99.94 | Pt3 | NC_075446.1 | PZ797561 |
| Bocavirus | NT | 0.00 | 901272 | 0.00 | Pt3 |  |  |
| Adenovirus | 22.82 | 11.24 | 4636182 | 98.02 | Pt4 | AC_000017.1 | PZ795375 |
| Coronavirus | 24.54 | 0.06 | 4636182 | 74.03 | Pt4 | NC_006213.1 |  |
| Bocavirus | NT | 0.00 | 4636182 | 0.00 | Pt4 |  |  |
| Adenovirus | 34.85 | 0.00 | 2150970 | 0.00 | Pt5 |  |  |
| RSV | 19.18 | 5.12 | 2150970 | 99.71 | Pt5 | NC_001781.1 | PZ797566 |
| Enterovirus | ND | 0.00 | 2150970 | 0.00 | Pt5 |  |  |
| Parainfluenzavirus | NT | 5.45 | 60868 | 98.65 | Pt6 | NC_075446.1 | PZ797567 |
| RSV | 29.6 | 4.04 | 60868 | 98.17 | Pt6 | NC_001781.1 |  |
| Parainfluenzavirus | NT | 1.21 | 1508374 | 94.04 | Pt7 | NC_021928.1 |  |
| Metapneumovirus | ND | 1.02 | 1508374 | 65.08 | Pt7 | NC_039199.1 |  |
| Coronavirus | 31.26 | 0.35 | 958358 | 64.56 | Pt8 | NC_006213.1 |  |
| Metapneumovirus | 31.43 | 0.00 | 3389958 | 26.92 | Pt9 | NC_039199.1 |  |
| Influenza | ND | 0.00 | 3389958 | 0.00 | Pt9 |  |  |
| Coronavirus | 24.44 | 0.11 | 3179242 | 70.46 | Pt10 | NC_006213.1 |  |
| Enterovirus | ND | 0.00 | 3179242 | 0.00 | Pt10 |  |  |
| Parainfluenzavirus | NT | 0.59 | 233868 | 81.56 | Pt11 | NC_021928.1 |  |
| Enterovirus | 26.36 | 0.00 | 233868 | 0.00 | Pt11 |  |  |
| Enterovirus | 19.64 | 0.02 | 799470 | 54.05 | Pt12 | NC_038308.1 |  |
| Influenza | ND | 0.00 | 799470 | 0.00 | Pt12 |  |  |
| Adenovirus | ND | 0.00 | 372472 | 0.00 | Pt13 |  |  |
| Enterovirus | ND | 0.00 | 372472 | 0.00 | Pt13 |  |  |
| RSV | ND | 0.00 | 372472 | 0.00 | Pt13 |  |  |
| Metapneumovirus | 23.1 | 66.94 | 110422 | 93.37 | Pt14 | NC_039199.1 |  |
| Coronavirus | ND | 0.00 | 110422 | 0.00 | Pt14 |  |  |
| RSV | 19.37 | 33.51 | 904598 | 99.99 | Pt15 | NC_001781.1 | PZ797550 |
| Coronavirus | ND | 0.00 | 904598 | 0.00 | Pt15 |  |  |
| Parainfluenzavirus | NT | 1.83 | 141864 | 78.48 | Pt16 | NC_021928.1 |  |
| Adenovirus | ND | 0.00 | 141864 | 0.00 | Pt16 |  |  |
| Enterovirus | 24.94 | 0.00 | 2118058 | 6.24 | Pt17 | NC_001472.1 |  |
| Parainfluenzavirus | NT | 0.00 | 2118058 | 0.00 | Pt17 |  |  |
| RSV | 23.39 | 6.80 | 1729104 | 99.89 | Pt18 | NC_001803.1 | PZ797551 |
| Parainfluenzavirus | NT | 0.00 | 1729104 | 0.00 | Pt18 |  |  |
| Parainfluenzavirus | NT | 0.60 | 4112806 | 99.73 | Pt19 | NC_075446.1 | PZ797552 |
| Metapneumovirus | 32.41 | 0.02 | 4112806 | 99.80 | Pt19 | NC_039199.1 |  |
| RSV | 21.7 | 19.94 | 253230 | 98.71 | Pt20 | NC_001803.1 | PZ797554 |
| Parainfluenzavirus | NT | 0.00 | 253230 | 0.00 | Pt20 |  |  |
| Enterovirus | ND | 0.00 | 253230 | 0.00 | Pt20 |  |  |
| Enterovirus | 32.69 | 0.00 | 307700 | 0.00 | Pt21 |  |  |
| RSV | 23.47 | 11.14 | 307700 | 98.29 | Pt21 | NC_001803.1 | PZ797555 |
| Bocavirus | NT | 0.00 | 307700 | 0.00 | Pt21 |  |  |
| Parainfluenzavirus | NT | 0.00 | 349662 | 0.00 | Pt22 |  |  |
| Enterovirus | ND | 0.00 | 349662 | 0.00 | Pt22 |  |  |
| Enterovirus | 24.51 | 0.00 | 1862126 | 0.00 | Pt23 |  |  |
| Adenovirus | ND | 0.00 | 1862126 | 0.00 | Pt23 |  |  |
| Coronavirus | ND | 0.00 | 1862126 | 0.00 | Pt23 |  |  |
| Coronavirus | 17.44 | 42.88 | 2617472 | 99.90 | Pt24 | NC_006213.1 | PZ797556 |
| Parainfluenzavirus | NT | 0.00 | 2617472 | 0.00 | Pt24 |  |  |
| Coronavirus | 22.78 | 13.39 | 623286 | 99.06 | Pt25 | NC_006213.1 | PZ797557 |
| Bocavirus | NT | 0.00 | 623286 | 0.00 | Pt25 |  |  |
| Enterovirus | 32.14 | 0.00 | 326372 | 0.00 | Pt26 |  |  |
| RSV | 26.57 | 0.41 | 326372 | 90.21 | Pt26 | NC_001781.1 |  |
| Coronavirus | ND | 0.00 | 326372 | 0.00 | Pt26 |  |  |
| Coronavirus | 34.49 | 0.00 | 1143854 | 0.00 | Pt27 |  |  |
| RSV | 18.32 | 27.14 | 1143854 | 99.78 | Pt27 | NC_001781.1 | PZ797558 |
| RSV | 23.49 | 4.99 | 3861930 | 99.70 | Pt28 | NC_001781.1 | PZ797559 |
| Bocavirus | NT | 0.00 | 52864 | 0.00 | Pt28 |  |  |
| Parainfluenzavirus | NT | 7.28 | 1830220 | 99.90 | Pt29 | NC_075446.1 | PZ797560 |
| Enterovirus | 28.93 | 0.00 | 1830220 | 0.00 | Pt29 |  |  |
| Metapneumovirus | 21.56 | 29.74 | 226422 | 93.63 | Pt30 | NC_039199.1 |  |
| Bocavirus | NT | 0.00 | 226422 | 0.00 | Pt30 |  |  |
| Enterovirus | 31.46 | 0.00 | 1110998 | 0.00 | Pt31 |  |  |
| RSV | 25 | 2.53 | 1110998 | 97.14 | Pt31 | NC_001803.1 |  |
| Parainfluenzavirus | NT | 0.00 | 1110998 | 0.00 | Pt31 |  |  |
| RSV | 14.56 | 78.66 | 1606370 | 100.00 | Pt32 | NC_001781.1 |  |
| Parainfluenzavirus | NT | 0.00 | 1606370 | 0.00 | Pt32 |  |  |
| Parainfluenzavirus | NT | 2.06 | 2598004 | 99.92 | Pt33 | NC_075446.1 | PZ797562 |
| Adenovirus | ND | 0.00 | 2598004 | 0.00 | Pt33 |  |  |
| Bocavirus | NT | 59.66 | 23474 | 96.72 | Pt34 | NC_007455.1 | PZ795376 |
| Enterovirus | 32.03 | 0.00 | 23474 | 0.00 | Pt34 |  |  |
| Parainfluenzavirus | NT | 0.00 | 23474 | 0.00 | Pt34 |  |  |
| Coronavirus | 20.2 | 3.40 | 552788 | 98.57 | Pt35 | NC_005831.2 | PZ797563 |
| Adenovirus | ND | 0.00 | 552788 | 0.00 | Pt35 |  |  |
| Enterovirus | ND | 0.00 | 552788 | 0.00 | Pt35 |  |  |
| Metapneumovirus | 23.5 | 2.81 | 1393176 | 95.00 | Pt36 | NC_039199.1 |  |
| RSV | 20.71 | 30.79 | 1393176 | 99.38 | Pt36 | NC_001803.1 | PZ797564 |
| Parainfluenzavirus | NT | 0.06 | 1064218 | 76.74 | Pt37 | NC_075446.1 |  |
| Enterovirus | 26.05 | 0.00 | 1064218 | 0.00 | Pt37 |  |  |
| Bocavirus | NT | 0.00 | 1064218 | 0.00 | Pt37 |  |  |
| Bocavirus | NT | 0.22 | 320602 | 88.56 | Pt38 | NC_007455.1 |  |
| Coronavirus | ND | 0.00 | 320602 | 0.00 | Pt38 |  |  |
| Adenovirus | 32.04 | 0.00 | 1862822 | 0.00 | Pt39 |  |  |
| Enterovirus | 32.08 | 0.00 | 1862822 | 0.00 | Pt39 |  |  |
| Bocavirus | NT | 58.03 | 1862822 | 100.00 | Pt39 | NC_007455.1 |  |
| Parainfluenzavirus | NT | 0.03 | 731934 | 50.52 | Pt40 | NC_075446.1 |  |
| Enterovirus | 32.85 | 0.00 | 731934 | 1.75 | Pt40 | NC_001617.1 |  |
| Bocavirus | NT | 0.00 | 731934 | 0.00 | Pt40 |  |  |
| Coronavirus | 32.55 | 0.00 | 225912 | 0.00 | Pt41 |  |  |
| Adenovirus | 31.3 | 0.82 | 225912 | 55.07 | Pt41 | AC_000008.1 |  |
| Bocavirus | NT | 0.16 | 2455968 | 95.96 | Pt42 | NC_007455.1 | PZ795377 |
| Enterovirus | ND | 0.00 | 2455968 | 0.00 | Pt42 |  |  |
| Parainfluenzavirus | NT | 0.33 | 356522 | 83.69 | Pt43 | NC_021928.1 |  |
| Enterovirus | 29.53 | 0.00 | 356522 | 0.00 | Pt43 |  |  |
| RSV | 21.98 | 7.21 | 356522 | 98.75 | Pt43 | NC_001803.1 | PZ797565 |
| Coronavirus | 25.16 | 12.37 | 142426 | 96.64 | Pt44 | NC_002645.1 |  |
| Enterovirus | ND | 0.00 | 142426 | 0.00 | Pt44 |  |  |
| Enterovirus | 28.49 | 0.00 | 335910 | 2.17 | Pt45 | NC_009996.1 |  |
| RSV | ND | 0.00 | 335910 | 0.00 | Pt45 |  |  |
| Influenza | 34.2 | 0.00 | 376030 | 0.00 | Pt46 |  |  |
| Enterovirus | 27.69 | 0.00 | 76920 | 0.00 | Pt47 |  |  |
| Metapneumovirus | ND | 0.00 | 76920 | 0.00 | Pt47 |  |  |
| Enterovirus | 27.3 | 0.00 | 328936 | 0.00 | Pt48 |  |  |
| Adenovirus | ND | 0.00 | 328936 | 0.00 | Pt48 |  |  |
| Coronavirus | ND | 0.00 | 328936 | 0.00 | Pt48 |  |  |
| RSV | 30.87 | 0.23 | 172400 | 54.79 | Pt49 | NC_001781.1 |  |
| Metapneumovirus | ND | 0.00 | 172400 | 0.00 | Pt49 |  |  |
| Enterovirus | 28.23 | 0.00 | 442088 | 0.00 | Pt50 |  |  |
| Coronavirus | ND | 0.00 | 442088 | 0.00 | Pt50 |  |  |
| Parainfluenzavirus | NT | 0.00 | 442088 | 0.00 | Pt50 |  |  |
| Coronavirus | ND | 0.00 | 121580 | 0.00 | Pt51 |  |  |
| Metapneumovirus | ND | 0.00 | 121580 | 0.00 | Pt51 |  |  |
| Enterovirus | 33.62 | 0.00 | 1739066 | 0.00 | Pt52 |  |  |
| RSV | ND | 0.00 | 1739066 | 0.00 | Pt52 |  |  |
| Adenovirus | ND | 0.00 | 29800 | 0.00 | Pt53 |  |  |
| Coronavirus | ND | 0.00 | 29800 | 0.00 | Pt53 |  |  |
| Enterovirus | 32.62 | 0.00 | 349790 | 0.00 | Pt54 |  |  |
| Adenovirus | ND | 0.00 | 349790 | 0.00 | Pt54 |  |  |
| Metapneumovirus | 32.76 | 0.02 | 861668 | 43.93 | Pt55 | NC_039199.1 |  |
| Parainfluenzavirus | NT | 0.00 | 861668 | 0.00 | Pt55 |  |  |
| Enterovirus | ND | 0.00 | 861668 | 0.00 | Pt55 |  |  |
| Enterovirus | 28.38 | 0.00 | 52864 | 0.00 | Pt56 |  |  |
| Adenovirus | ND | 0.00 | 52864 | 0.00 | Pt56 |  |  |
| Parainfluenzavirus | NT | 0.00 | 52864 | 0.00 | Pt56 |  |  |
| Coronavirus | 32.76 | 0.00 | 330322 | 0.00 | Pt57 |  |  |
| Enterovirus | 34.28 | 0.00 | 330322 | 0.00 | Pt57 |  |  |
| Coronavirus | 24.64 | 0.00 | 265536 | 0.00 | Pt58 |  |  |
| Enterovirus | 23.46 | 0.00 | 265536 | 0.00 | Pt58 |  |  |
| Enterovirus | 24.64 | 0.00 | 4877448 | 0.00 | Pt59 |  |  |
| Parainfluenzavirus | NT | 0.00 | 4877448 | 0.00 | Pt59 |  |  |
| Influenza | 34.85 | 0.00 | 4214230 | 0.00 | Pt60 |  |  |
| Adenovirus | ND | 0.00 | 4214230 | 0.00 | Pt60 |  |  |
| Enterovirus | ND | 0.00 | 4214230 | 0.00 | Pt60 |  |  |
| Adenovirus | 34.88 | 0.00 | 2751164 | 0.00 | Pt61 |  |  |
| RSV | 17.52 | 17.03 | 2751164 | 99.99 | Pt61 | NC_001781.1 |  |

**Supplementary Table 2:** In-house Real-time Semi-quantitative assays.

| Assay | Respiratory virus | Primer/Probe | Concentration (nM) | Sequence (5’ to 3’) | Cycling conditions |
| --- | --- | --- | --- | --- | --- |
| 1 | Adenovirus | Adeno Forward | 900 | GCC CCA RTG GKC NTA CAT GCA CAT C | 25°C for 2 minutes  53°C for 10 minutes  95°C for 2 minutes  40 cycles    95°C for 3 seconds  60°C for 30 seconds |
|  |  | Adeno Reverse | 800 | GCC ACI GTG GGR TTY CTR AAC TT |  |
|  |  | Adeno Probe | 275 | Cy5.5-TGC ACC AGA CCC GGR CTC AGR TAC TCC GA-QSY2 |  |
|  | Enterovirus | Enterovirus Forward | 600 | GCC CCT GAA TGC GGC TAA T |  |
|  |  | Enterovirus Reverse | 800 | AAA CAC GGA CAC CCA AAG TA |  |
|  |  | Enterovirus Probe | 150 | 6FAM- TCT GYR GCG GAA CCG ACT-NFQ-MGB |  |
|  |  | Rhinovirus Probe | 150 | VIC-TCY GGG AYG GGA CCR ACT A-NFQ-MGB |  |
| 2 | Influenza  A/B  H1/H3 | AM Forward | 900 | GAG TCT TCT AAC MGA GGT CGA AAC GTA |  |
|  |  | AM Reverse | 900 | GGG CAC GGT GAG CGT RAA |  |
|  |  | BNP Forward | 400 | GCA GCT CTG ATG TCC ATC AAG CT |  |
|  |  | BNP Reverse | 900 | CAG CTT GCT TGC TTA RAG CAA TAG GTC T |  |
|  |  | AH1pdm09 10Y11Y Forward | 900 | TTA CCA GAT YYT GGC RAT CTA YT |  |
|  |  | AH1pdm09 17Y Reverse | 800 | CCA GGG AGA CTA SCA RYA CCA |  |
|  |  | AH3 Forward | 600 | TGG GAC CTT TTY GTT GAA MG |  |
|  |  | AH3 Reverse | 600 | CGG ATG AGG CAA CTA GTG AYC TA |  |
|  |  | AM49 | 250 | JUN-TCA GGC CCC CTC AAA GCC GAG-QSY |  |
|  |  | BNP | 30 | ABY-CCA GAY CTG GTC ATY GGA GCC CAA AAC TG-QSY |  |
|  |  | AH1(3T5Y12Y) | 225 | 6FAM-ACT GYY GCC AGY TC-MGBNFQ |  |
|  |  | AH3 (8Y) | 250 | VIC-CCW ACA GYA RCT GTT AYC-MGBNFQ |  |
| 3 | RSV A/B | RSV (For) | 600 | MAG AGG KGG CAG TAG AGT TGA |  |
|  |  | RSV (Rev) | 800 | CCT GMA CCA TAG GCA TTC AT |  |
|  |  | RSV A | 200 | FAM-AGG GAT TTT TGC AGG ATT G-BHQ1 |  |
|  |  | RSV B | 250 | Quasar705-AGG AAT CTT TGC AGG ATT G-BHQ2 |  |
|  | Metapneumovirus | hMPV A (For) | 300 | CAG CAC CAG ACA CAC CTA TAA T |  |
|  |  | hMPV A (Rev) | 600 | TGC ATC ACT TAG TAC ACG GTT AG |  |
|  |  | hMPV A | 150 | ABY-AGT GGG ATT AGA GAC CAC AGT CAG AAG A-QSY |  |
|  |  | hMPV B (For) | 300 | GGG TGT CAT TGC CAG ATC AT |  |
|  |  | hMPV B (Rev) | 300 | CCA GAT TCA GGA CCC ATT TCT C |  |
|  |  | hMPV B | 150 | ABY-AGG GCA TGT ATC TGT GCA AGC TGA-QSY |  |
| 4 | SARS-CoV-2 | E Sarbeco Forward | 600 | ACA GGT ACG TTA ATA GTT AAT AGC GT |  |
|  |  | E Sarbeco Reverse | 600 | ATA TTG CAG CAG TAC GCA CAC A |  |
|  |  | E Sarbeco Probe | 200 | ABY-ACA CTA GCC ATC CTT ACT GCG CTT CG-QSY |  |
|  |  | ORF1ab Forward | 600 | CCC TGT GGG TTT TAC ACT TAA |  |
|  |  | ORF1ab Reverse | 800 | ACG ATT GTG CAT CAG CTG A |  |
|  |  | ORF1ab Probe | 200 | FAM-CCG TCT GCG GTA TGT GGA AAG GTT ATG-BHQ2 |  |

**Supplementary table 3:** Versions and parameters of software used.

| Software | Version | Parameters |
| --- | --- | --- |
| Burrows Wheeler Aligner | 0.7.13 | mem -t 4 |
| metaSPAdes | 3.11.1 | -k 21,33, 55, 77, 99, 127 |
| Trimmomatic | 0.36 | LEADING: 20, TRAILING: 20, SLIDINGWINDOW:5:20, MINLEN:75 |
| SAMtools | 1.3 |  |
| BLAST | 2.2.27 | -max_target_seqs 1, -outfmt “10 sseqid” |
| QuasiBAM | 2.8 | -vb on |

**Supplementary table 4:** Positive percentage agreement and overall percent agreement for each method (NT=Not tested, grey=data unavailable, CI=Confidence interval).

| Reference method | Test method | Target | Positive percent agreement (PPA) | 95% CI | Negative percent agreement (NPA) | 95% CI | Overall percent agreement (OPA) | 95% CI |
| --- | --- | --- | --- | --- | --- | --- | --- | --- |
| WGS | ePlex | Adenovirus | 100.00 | 34.24-100.00 |  |  | 12.50 | 3.50-36.02 |
|  |  | Bocavirus | 100.00 | 51.01-100.00 |  |  | 33.33 | 13.81-60.94 |
|  |  | Coronavirus | 100.00 | 70.09-100.00 |  |  | 40.91 | 23.26-61.27 |
|  |  | Enterovirus | 100.00 | 51.01-100.00 |  |  | 11.76 | 4.67-26.62 |
|  |  | Influenza |  |  |  |  | 0.00 | 0.00-48.99 |
|  |  | Metapneumovirus | 100.00 | 64.57-100.00 |  |  | 70.00 | 39.68-89.22 |
|  |  | Parainfluenza virus | 100.00 | 74.12-100-00 |  |  | 47.83 | 29.24-67.04 |
|  |  | RSV | 100.00 | 80.64-100.00 |  |  | 80.00 | 58.40-91.93 |
|  |  | All viruses | 100.00 | 93.24-100-00 |  |  | 37.04 | 30.02-45.81 |
| RT-qPCR | ePlex | Adenovirus | 100.00 | 56.55-100.00 |  |  | 31.25 | 14.16-55.60 |
|  |  | Bocavirus | NT | | | | | |
|  |  | Coronavirus | 100.00 | 77.19-100.00 |  |  | 59.09 | 38.73-76.74 |
|  |  | Enterovirus | 100.00 | 74.12-100.00 |  |  | 32.35 | 19.13-49.16 |
|  |  | Influenza | 100.00 | 34.24-100.00 |  |  | 50.00 | 15.00-85.00 |
|  |  | Metapneumovirus | 100.00 | 60.97-100.00 |  |  | 60.00 | 31.27-83.18 |
|  |  | Parainfluenza virus | NT | | | | | |
|  |  | RSV | 100.00 | 81.57-100.00 |  |  | 85.00 | 63.96-94.76 |
|  |  | All viruses | 100.00 | 93.36-100.00 |  |  | 52.95 | 41.56-60.26 |
| RT-qPCR | WGS | Adenovirus | 40.00 | 11.76-76.93 | 100.00 | 74.12-100.00 | 81.25 | 56.99-93.41 |
|  |  | Bocavirus | NT | | | | | |
|  |  | Coronavirus | 69.23 | 42.37-87.32 | 100.00 | 70.09-100.00 | 81.82 | 61.48-92.69 |
|  |  | Enterovirus | 17.39 | 6.98-37.14 | 100.00 | 74.12-100.00 | 44.12 | 28.88-60.55 |
|  |  | Influenza | 0.00 | 0.00-65.76 | 100.00 | 34.24-100.00 | 50.00 | 15.00-85.00 |
|  |  | Metapneumovirus | 100.00 | 60.97-100.00 | 75.00 | 30.06-95.44 | 90.00 | 59.59-98.21 |
|  |  | Parainfluenza virus | NT | | | | | |
|  |  | RSV | 94.12 | 73.02-98.95 | 100.00 | 43.85-100.00 | 95.00 | 76.39-99.11 |
|  |  | All viruses | 56.06 | 44.08-67.37 | 97.37 | 87.12-99.56 | 73.70 | 62.48-79.40 |
